# Author Correction: Multivariate EEG analyses support high-resolution tracking of feature-based attentional selection

**DOI:** 10.1038/s41598-018-29326-5

**Published:** 2018-07-20

**Authors:** Johannes Jacobus Fahrenfort, Anna Grubert, Christian N. L. Olivers, Martin Eimer

**Affiliations:** 1 Department of Experimental and Applied Psychology & Institute for Brain and Behavior Amsterdam (iBBA), Vrije Universiteit, Amsterdam, The Netherlands; 2 Department of Psychology, Durham University, Durham, UK; 3 Department of Psychological Sciences, Birkbeck, University of London, London, UK

Correction to: *Scientific Reports* 10.1038/s41598-017-01911-0, published online 15 May 2017

This Article contains an error in the legend of Figure 6.

“Decoding accuracy in Experiment 2. (**A**) 8-way classification accuracy of target position (chance = 0.125). The black line reflects significance at p < 10^−3^ (cluster based permutation test), shaded area is +/− s.e.m. Peak at 264 ms. Note again the similarity in temporal evolution with the N2pc (Fig. 3) and decoding accuracies of Experiment 1 (Fig. 4), although plausibly peaking slightly later because of the increase in the number of items on the screen. (**B**) Classsification accuracy per quadrant (chance = 0.5). (**C**) Separate N2pc components were computed for left versus right target objects at each of the four vertical elevations, indicated by color. Colored sections indicate p < 10^−3^ (cluster based permutation test).”

should read:

“Comparing the N2pc to decoding accuracy in Experiment 2. (**A**) Average N2pc for left and right targets in Experiment 2, computed using PO7 and PO8 (see Methods). Peak at 260 ms. (**B**) 8-way classification accuracy of target position in Experiment 2 (chance = 0.125). Peak at 264 ms. Note again the similarity in temporal evolution of decoding accuracy in panel B when compared to the temporal evolution of the N2pc in panel A. (**C**) Separate N2pc components at each of the four vertical elevations, indicated by color. Thick black or colored lines reflect significance at p < 10^−3^ (cluster based permutation test), shaded areas are +/− s.e.m.”

